# Wearable Sensor-Based Residual Multifeature Fusion Shrinkage Networks for Human Activity Recognition

**DOI:** 10.3390/s24030758

**Published:** 2024-01-24

**Authors:** Fancheng Zeng, Mian Guo, Long Tan, Fa Guo, Xiushan Liu

**Affiliations:** School of Electronics and Information, Guangdong Polytechnic Normal University, Guangzhou 510660, China; z_fancheng@163.com (F.Z.); deavontan@gmail.com (L.T.); wy_guofa@163.com (F.G.);

**Keywords:** human activity recognition (HAR), multifeature extraction, feature fusion, attention mechanism, feature selection

## Abstract

Human activity recognition (HAR) based on wearable sensors has emerged as a low-cost key-enabling technology for applications such as human–computer interaction and healthcare. In wearable sensor-based HAR, deep learning is desired for extracting human active features. Due to the spatiotemporal dynamic of human activity, a special deep learning network for recognizing the temporal continuous activities of humans is required to improve the recognition accuracy for supporting advanced HAR applications. To this end, a residual multifeature fusion shrinkage network (RMFSN) is proposed. The RMFSN is an improved residual network which consists of a multi-branch framework, a channel attention shrinkage block (CASB), and a classifier network. The special multi-branch framework utilizes a 1D-CNN, a lightweight temporal attention mechanism, and a multi-scale feature extraction method to capture diverse activity features via multiple branches. The CASB is proposed to automatically select key features from the diverse features for each activity, and the classifier network outputs the final recognition results. Experimental results have shown that the accuracy of the proposed RMFSN for the public datasets UCI-HAR, WISDM, and OPPORTUNITY are 98.13%, 98.35%, and 93.89%, respectively. In comparison with existing advanced methods, the proposed RMFSN could achieve higher accuracy while requiring fewer model parameters.

## 1. Introduction

There has long been a continual aspiration to leverage technological advancements for the understanding and analysis of human behavior, aiming at improving both quality of life and working environments. In recent years, motivated by the growing development of the Internet of Things (IoT) and artificial intelligence (AI) technologies, AI-based human activity recognition (HAR) has been regarded as pivotal technology, capturing widespread interest [1]. AI-based HAR would have substantial potential and significance in multiple domains such as human–computer interaction [2,3], health monitoring [4,5], and smart homes [6,7].

Considering the type of human activity data, HAR technology can be categorized as either vision-based HAR or sensor-based HAR [8]. The former relies on visual information obtained from images or videos to analyze visual features such as human postures, movements, and shapes [9,10]. The latter relies on wearable sensors or environmental perception sensors to capture information related to human motion, posture, and physiological signals for analysis [11,12,13]. Vision-based HAR has the significant advantage of acquiring large-scale training data, since images and video data can intuitively capture activities. However, it is essential to note that changes in perspective and lighting conditions may introduce disturbances in appearance and features, thereby adversely affecting the accuracy of activity recognition. Additionally, human activities often occur in complex and dynamic environmental settings, sometimes involving multiple subjects simultaneously. In such cases, factors such as background interference and mutual occlusion of subjects may increase the complexity of recognition tasks. Moreover, privacy concerns present a challenge for vision-based methods. In contrast, the wearable sensor-based HAR method is less susceptible to environmental variations. In addition, since only wearable sensor data are collected, it is also less vulnerable to security attacks and privacy issues. In recent years, as sensor technology and the IoT have rapidly advanced, an increasing number of researchers are leaning toward adopting wearable sensor-based HAR methods to address the challenges and limitations present in vision-based approaches [14].

Traditional HAR approaches utilize machine learning algorithms for classification, such as support vector machines (SVMs) [15,16], random forest [17,18], k-nearest neighbors (KNN) [19], and hidden Markov models (HMM), among others [20,21]. However, these methods have certain limitations in deep feature extraction, especially for complex action recognition tasks. Their performance is often restricted, and they require domain knowledge and manual feature extraction. In recent years, with the rise of deep learning technologies, deep neural networks have excelled in the HAR field and gradually become the mainstream approach [22]. Deep neural networks possess powerful feature learning capabilities, allowing them to automatically extract high-level features from raw data without the need for extensive manual feature engineering. This advantage enables them to better handle the recognition of complex motion patterns and various environmental conditions associated with human activity recognition tasks.

**Figure 1:**
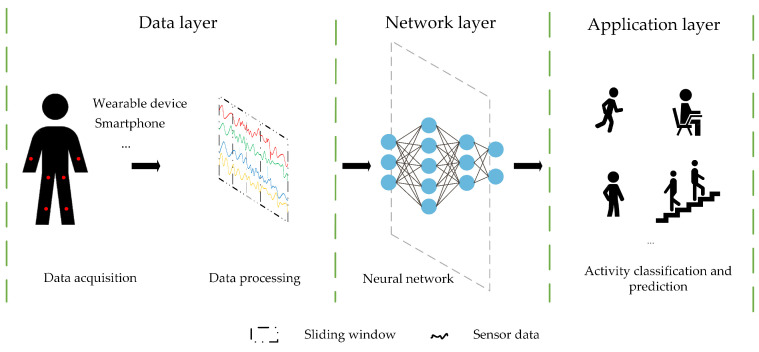
A sensor-based human activity recognition system using deep neural networks.

As depicted in Figure 1, a sensor-based deep learning HAR system generally consists of the data, network, and application layers. Current research trends primarily focus on data processing at the data layer and adjustments to the neural network structure, aiming at optimizing the model’s learning capabilities to enhance HAR performance. In data processing, researchers have proposed various methods, including dynamic sliding window segmentation [23], an adaptive batch size [24], and a wavelet transform, among others [25,26]. Although these methods aid in handling sensor data and extracting valuable features, their complexity and additional computational costs should not be overlooked.

Faced with diverse and complex activity data, a deep network only consists of a convolutional neural network or recurrent neural network like long short-term memory (LSTM) and gated Rrecurrent units (GRUs), which have shown limitations in recent years. Consequently, researchers are increasingly inclined to use hybrid network structures [27]. These hybrid networks combine different types of neural network components to better adapt to diverse data and tasks. Kocşar et al. introduced a 2D CNN-LSTM hybrid model, which utilizes a dual-branch structure to extract diverse features from wearable sensor data [28]. Similarly, Venkatachalam et al. proposed a bimodal hybrid classifier with 1D CNN-LSTM to enhance HAR performance [29]. Han et al. proposed a heterogeneous convolutional architecture that can generate activity features with more discriminative power by simultaneously employing different convolution kernel sizes to capture different time scales, and it can be integrated into existing models to improve accuracy [30].

Furthermore, with the rise of attention mechanisms, integrating them into hybrid models has become a significant trend. Attention mechanisms allow models to focus more on the important parts of the data, thereby enhancing HAR accuracy and efficiency [31]. Mim et al. proposed an efficient recognition model [32] that utilizes GRUs with an attention mechanism to extract temporal features and inception with a convolutional block attention module (CBAM) for spatial feature extraction, and it includes residual connections to address gradient vanishing issues. Ding et al. introduced a deep convolutional network hybrid model that extracts spatiotemporal features through three convolution layers and three parallel pathways [33]. The convolution layers capture the spatial features, while the parallel pathways, consisting of LSTM and GRUs, extract the temporal features. A channel attention mechanism, SENet, is introduced after the convolution layers to improve performance. Wang et al. proposed a multi-feature extraction model composed of Bi-GRU, a CNN, and ResNet, with different attention mechanisms allocated to each feature extraction layer [34]. Similarly, Sun et al. presented a multifeature extraction framework consisting of a capsule block and two GRU layers [35]. Each GRU layer in this framework incorporates different attention mechanisms, effectively enhancing recognition performance.

Although existing research has been able to effectively extract various features from sensor data and achieve good performance, there is still room for improvement in feature fusion and selection. In particular, for the issues of selecting important feature channels from the extracted features based on different activity types, existing works have not extensively addressed this issue. In this paper, we propose a a residual multifeature fusion shrinkage network to address the above issues in wearable-based HAR. Our main contributions are as follows:

- The residual multifeature fusion shrinkage network (RMFSN) is proposed for wearable-based HAR, which consists of a temporal feature extraction block (TFEB), multi-scale feature extraction block (MSFEB), channel attention shrinkage block (CASB), and a classifier network. In the RMFSN, a lightweight temporal attention mechanism is used for enhancing HAR accuracy. In addition, a channel attention shrinkage network is designed for adaptively selecting the most relevant features based on activity types, which further improves the accuracy and generalization. The RMFSN can effectively integrate various features extracted from sensor data while having a relatively small number of model parameters, making it suitable for potential applications in resource-constrained environments.
- Data preprocessing for learning is carried out. Since data are subject to the issues of missing values and anomalies, it cannot be directly used in deep neural networks for learning. Accordingly, we have proposed data cleaning methods for the investigated public datasets such as UCI-HAR, WISDM and OPPORTUNITY, considering the special features of earch dataset.
- Experimental results have been conducted to demonstrate the efficiency of the proposed RMFSN in wearable-based HAR.

The remainder of this paper is organized as follows. Section 2 presents the detail of the proposed RMFSN and the features of public datasets for HAR. Section 3 provides the performance evaluation of the RMFSN through experiments with public datasets. Section 4 concludes the paper.

## 2. Methodology

**Figure 2:**
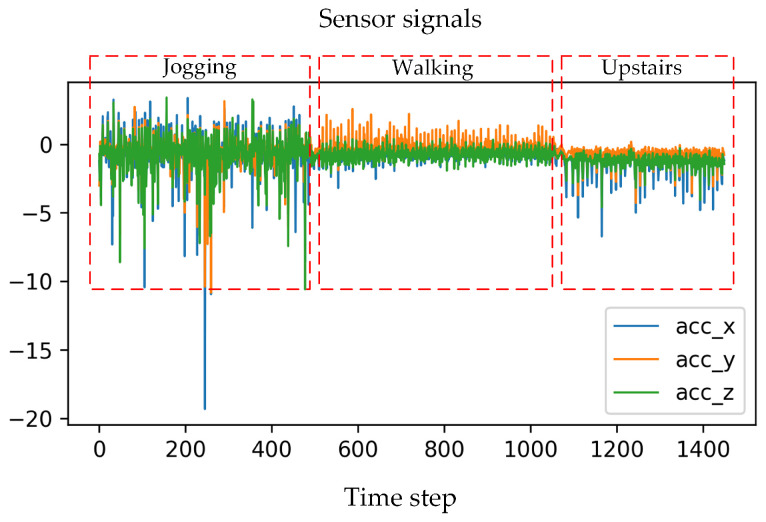
Sensor signals acquired from the accelerometer.

The task of HAR based on wearable sensors typically involves the analysis of IMU sensors, such as accelerometers and gyroscopes, to extract diverse features from sensor-generated time series data for the purpose of human activity classification and prediction. Figure 2 presents partial sensor signals collected by wearing accelerometers, encompassing three types of activities: jogging, walking, and upstairs. Obviously, different activities have distinct patterns in the sensor signals. Taking jogging as an example, the oscillations in the x, y, and z axes exhibit a noticeable amplitude due to the significant swinging motion of the body, surpassing the intensity observed during walking, as illustrated in Figure 2. This paper aims to design an HAR method (e.g., RMFSN) for recognizing human activity via effectively identifying the special sensor signal properties of human activities. The details are below.

### 2.1. The Proposed RMFSN

**Figure 3:**
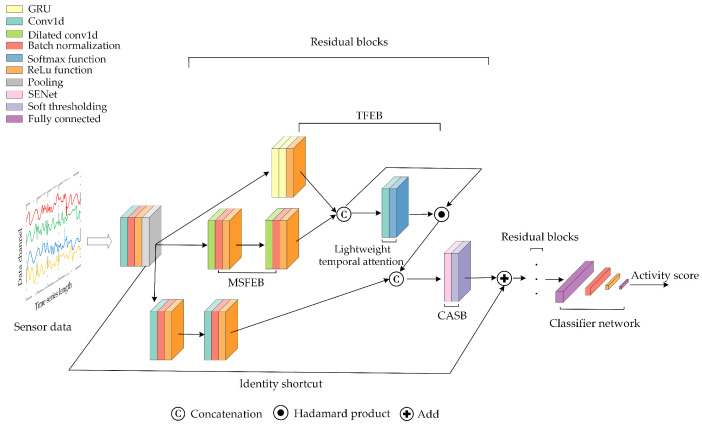
Architecture of the proposed model.

Based on a residual network, we designed an RMFSN for wearable-based HAR, which consists of a temporal feature extraction block (TFEB), a multi-scale feature extraction block (MSFEB), a channel attention shrinkage block (CASB), and a classifier network, as illustrated in Figure 3. In the RMFSN, we first extract the initial features from the raw data using a convolutional network. Notice that the input size of the convolutional network should adjust to the data dimension, which is determined by the channels and sampling frequencies of the HAR sensors. Different public datasets may use different HAR sensors, leading to distinct data dimensions. Then, the extracted features are simultaneously distributed to three distinct branches, consisting of TFEB, MSFEB, and a standard convolutional block, each dedicated to extracting distinct types of features. In particular, the MSFEB fuses the features extracted by the GRUs within the TFEB and integrates a lightweight temporal attention mechanism to assist in the extraction of temporal features. The features extracted from the previous three branches are consolidated and fed into the CASB. Then, the CASB intelligently selects the most crucial feature channels for each activity class using automatically learned thresholds. The selected crucial feature channels then perform weighted feature shrinkage via a soft threshold function. Finally, the shrunken features are mapped through the classifier network to produce scores for various activity categories.

It is worth noting that in the RMFSN, the convolutional layers are designed with only one dimension, the reason being that the one-dimensional convolutional neural network (1D-CNN) has been demonstrated to effectively capture local patterns and features in sensor time series data [36]. Additionally, the 1D-CNN require fewer parameters, and thus with the 1D-CNN, the model complexity and computation cost in the RMFSN can be explictly reduced.

The details of the main blocks of the RMFSN for wearable-based HAR are described below.

#### 2.1.1. TFEB

In our daily lives, human activities encompass various movements and actions across different time scales. Sensor signals may involve long-term dependencies, such as extended time intervals between the initiation and completion of a particular action. A comprehensive understanding of these temporal dependencies is crucial for enhancing the accuracy of HAR. In this paper, we constructed a TFEB consisting of two GRU networks and a lightweight attention mechanism to capture the long-term dependencies effectively. Within the GRU network, two key gate mechanisms are employed: the update gate and the reset gate. These gate mechanisms enable the GRUs to finely control the propagation and retention of crucial information within sensor data. In comparison with the LSTM network, the GRU network possesses a more concise parameter structure, contributing to a reduced risk of overfitting, especially in scenarios with limited dataset sizes [37]. Furthermore, the attention mechanism empowers the model to concentrate on pivotal time steps, fostering adaptability to diverse time scales of movements and activities and thereby improving the precision of HAR.

**Figure 4:**
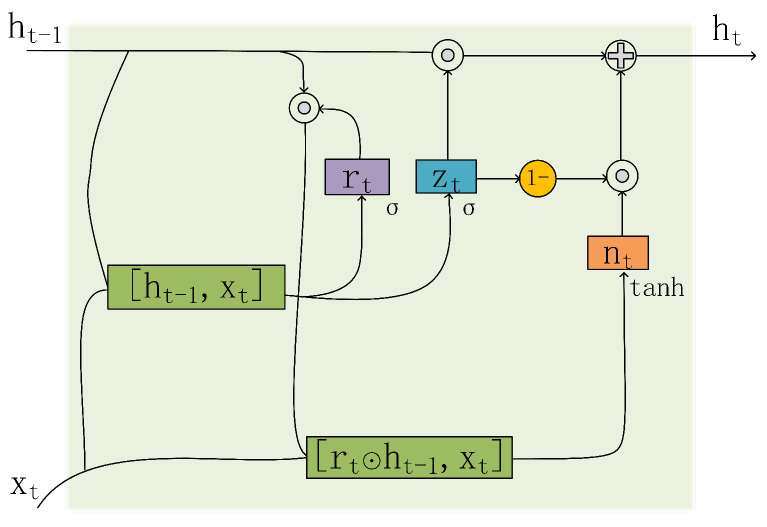
Structure of the GRUs.

As illustrated in Figure 4, the process through which the GRUs extract long-term correlated features from the sensor signals is as follows. Firstly, by computing the reset gate and the update gate, information flow is controlled based on the current input and the previous time step’s hidden state. The reset gate determines which old information to retain, and the update gate decides how much new information to retain. Next, by multiplying the reset gate by the previous time step’s hidden state and adding the current input, followed by nonlinear processing through the hyperbolic tangent function, a new memory candidate is generated. Subsequently, the update gate is used to determine how much old information to keep and is multiplied by the old hidden state, and the measured value of the update gate is added to the new memory, thus integrating old and new information to obtain the current time step’s hidden state. Finally, the current time step’s hidden state can be directly used as an output or passed to the next time step. The mathematical formulas associated with the above process are depicted in Equations (1)–(4) below:

(1) $$r_{t}=\sigma \left(W_{r}\left[\begin{array}{c} x_{t}\\ h_{t-1} \end{array}\right]+b_{r}\right),$$

(2) $$z_{t}=\sigma \left(W_{z}\left[\begin{array}{c} x_{t}\\ h_{t-1} \end{array}\right]+b_{z}\right),$$

(3) $$n_{t}=\tanh\left(W_{n}\left[\begin{array}{c} x_{t}\\ r_{t}⊙h_{t-1} \end{array}\right]+b_{n}\right),$$

(4) $$h_{t}=\left(1-z_{t}\right)⊙n_{t}+z_{t}⊙h_{t-1},$$

where $r_{t}$ represents the reset gate, $z_{t}$ is the update gate, $n_{t}$ is the learned new memory, and $h_{t}$ represents the hidden state at the current time step, while W and b denote the weight matrices and bias vectors for each branch, respectively.

#### 2.1.2. Lightweight Temporal Attention Mechanism

In this work, we have devised a relatively straightforward yet effective approach for implementing a temporal attention mechanism. We utilized one-dimensional convolutional layers with a kernel size of one, followed by a Softmax operation. While it may seem simple, within this context, it effectively models the degree of attention given to different time steps in the input sequence. Importantly, this mechanism introduces minimal additional computational cost and can be seamlessly integrated into other network structures.

The fundamental principle involves sliding a convolutional kernel along the time dimension and conducting local weighted linear combinations on different time steps of the input sequence. The convolutional kernel weights and aggregates data from various time steps, generating a new sequence where each time step is influenced by its neighbors. Subsequently, the Softmax function transforms the output of the convolutional layer into attention weights. Softmax scales these values to a range between 0 and 1, indicating the model’s attention to different time steps in the input sequence. Mathematically, this process can be represented as follows.

Assuming the input sequence is represented as $\left\{x_{1},x_{2},\ldots ,x_{i},\ldots ,x_{n}\right\}$, where $x_{i}$ represents the input data at different time steps, *n* is the length of the sequence. Initially, a one-dimensional convolutional kernel with a size of one, denoted as *w*, is used to perform local weighted linear combinations for the input sequence:

(5) $$c_{i}=w*x_{i},$$

where $c_{i}$ represents the output of the convolutional kernel at time step *i* and ∗ denotes the convolution operation.

Subsequently, the results of these local weighted linear combinations are combined to form a new sequence $\left\{c_{1},c_{2},\ldots ,c_{n}\right\}$. Following that, Softmax is used to transform the sequence into an attention weight sequence $\left\{a_{1},a_{2},\ldots ,a_{i},\ldots ,a_{n}\right\}$, where $a_{i}$ for i∈{1,2,…,n} is the degree of attention the model assigns to different time steps in the input sequence, which is defined as

(6) $$a_{i}=\frac{e^{c_{i}}}{\sum_{j=1}^{n}e^{c_{j}}}.$$

#### 2.1.3. MSFEB

GRUs are primarily used to capture the local dependency in sensor data. However, to capture dependency over a broader scope, increasing the depth of the GRUs inevitably leads to a proliferation of model parameters. In contrast, dilated convolution, which uses a dilation rate parameter to control the spacing inside the convolution kernel [38] and partially expand the model’s receptive field, enabling the extraction of more extensive features. Thus, it can effectively capture the multi-scale information while reducing the computational cost. Therefore, dilated convolution is particularly suitable for real-time HAR in edge devices or resource-constrained environments.

In the MSFEB, multiple layers of dilated convolutions are used with distinct dilation rates, and thus the MSFEB component can exhibit flexibility in handling various scales of human activities, such as rapid and slow movements. By combining the TFEB and MSFEB, our model is more flexible; that is, it is less susceptible to restrictions imposed by specific patterns.

**Figure 5:**
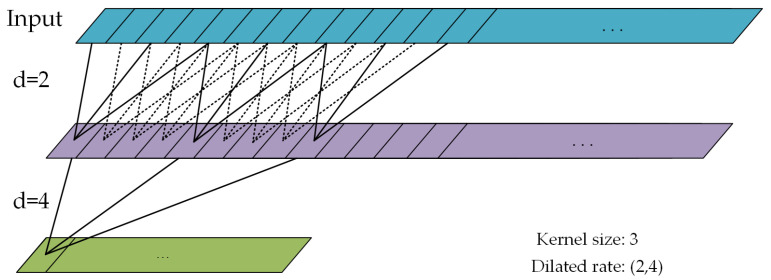
Multi-scale feature extraction structure of dilated convolution.

The dilated convolution-specific principle is depicted in Figure 5. The figure illustrates the structure of two layers of dilated convolutions, with a uniform kernel length of three, a convolution stride of one, zero padding, and dilation rates of two and four. It can be observed that each layer’s features are connected to multiple feature data from the previous layer, and each feature connection has a different time scale. By skillfully combining the dilation rates and convolution kernel parameters, deep features in the output can contain original information from different time scales. The range of information contained in the lower layer from the upper layer is referred to as the receptive field. The specific calculation formula is as follows:

(7) $$r_{i}=r_{i-1}+d\left(k-1\right)\times \prod_{n=1}^{i}s_{n-1},$$

where $r_{i}$ is the receptive field of the *i*th layer, *d* is the dilation rate, *k* denotes the convolution kernel size, and $s_{n-1}$ is the convolution stride of the (n−1)th layer.

#### 2.1.4. CASB

**Figure 6:**
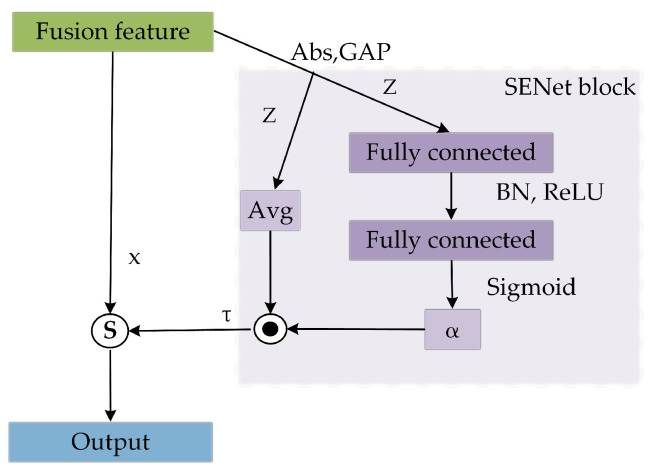
The structure of the CASB.

In HAR, some sensors may provide crucial action information, while others may contain less relevant data. For example, an accelerometer may capture rapid movements in a short time, while a gyroscope may be more suitable for capturing motion over a longer duration. Inspired by the DRSN proposed by Zhao et al. [39], we employed SENet [40] and soft thresholding to create the CASB for automatic selection of the essential feature channels. Although the sensor signal is processed in the preprocessing stage, it will inevitably be mixed with some noise. Soft thresholding can suppress these noises and make sparse the activation values to shrink the features. The structure, as shown in Figure 6, primarily involves SENet, which adaptively adjusts the weight of each channel in the feature map through learning channel relationships, thereby increasing the network’s ability to perceive important features.

SENet consists of two steps: squeeze and excitation. In the squeeze phase, global average pooling (GAP) is used to compress the feature values of each channel into a single numeric value. In the excitation phase, a small network is employed to learn the importance weight of each channel. This small network comprises two fully connected layers with ReLU activation in between, and the channel weights are normalized to a range between 0 and 1 using the sigmoid activation function, resulting in a weight vector. Originally, SENet directly multiplied the weight vector by the input to obtain weighted data. In our approach, we take the average absolute value of the feature vector obtained after pooling and multiply it by the weight vector to obtain a set of thresholds. These thresholds are used for subsequent soft thresholding. The specific calculation process can be represented using the following formulas:

(8) $$Z=\frac{1}{L}\sum_{i=1}^{L}x_{k,i},\;k=1,2,\ldots ,n$$

(9) $$\alpha =\sigma \left(W_{2}\mathrm{ReLU}\left(W_{1}Z\right)\right),$$

(10) $$\tau =\bar{\left|Z\right|}⊙\alpha ,$$

where Z represents the feature vector compressed through GAP across all data channels, *L* is the length of the input sequence, *n* is the number of feature channels, $x_{k,i}$ represents the value at the *i*th position of the *k*th channel sequence, α is the weight coefficient vector, σ represents the Sigmoid activation function, $W_{1}$ and $W_{2}$ are the weighted vectors for fully connected layers 1 and 2, respectively, ReLU(·) represents the ReLU activation function, $\bar{\left|Z\right|}$ is the average of *Z*, and τ is the learned threshold vector.

Then, a soft-thresholding function S(·), as described in Equation (11), is proposed to shrink the original HAR data *x* based on the the learned feature vector τ:

(11) $$S\left(x,\tau \right)=\left\{\begin{array}{cc} \mathrm{sign}(x)(x+\tau ), & x>\tau \\ \mathrm{sign}(x)(x-\tau ), & x<-\tau \\ 0, & \mathrm{otherwise} \end{array}\right.$$

#### 2.1.5. Classifier Network

**Figure 7:**
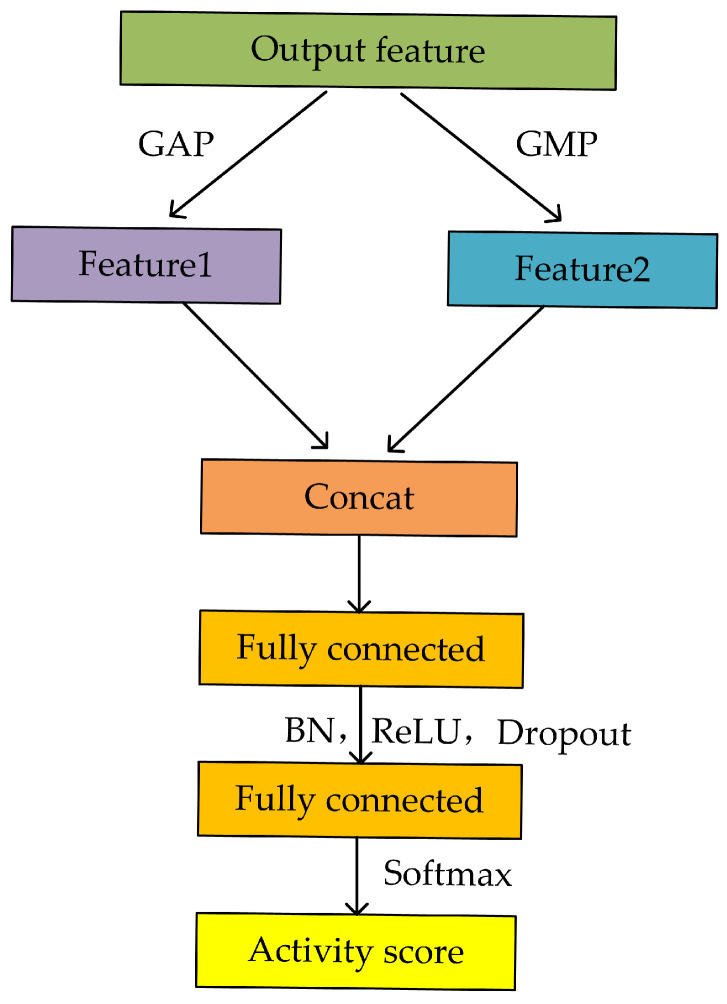
The structure of the classifier network.

To map the previously learned features to the final output results, a small classifier network is introduced in the last stage of the model. The specific structure of this classifier network is shown in Figure 7. To achieve better classification performance, a feature fusion process is added to the front end of the network. Firstly, important features are extracted from the output of the CASB through global average pooling and global max pooling (GMP) to obtain compressed key features. These two key features are then aggregated and passed to a fully connected layer for learning. To reduce the risk of overfitting, regularization techniques like dropout are introduced in the fully connected layer to reduce connections between some neurons. Finally, the SoftMax function is used to output the scores for different activity classes, with the activity having the highest score being the model’s predicted activity.

### 2.2. Dataset and Preprocessing

This paper considers three typical publicly available activity recognition datasets to evaluate the performance of our model, namely UCI-HAR [41], WISDM [42], and OPPORTUNITY [43]. These datasets are widely recognized and adopted in the academic community, ensuring that our model can be thoroughly evaluated across different backgrounds and application scenarios. The details of the investigated datasets and our preprocessing methods are as follows.

#### 2.2.1. Dataset Description

- UCI-HAR: The UCI-HAR dataset was collected from 30 volunteers aged between 19 and 48 who wore triaxial accelerometers and gyroscopes on their waists. The sensors recorded data along three axes, with acceleration and gyroscope readings. Each volunteer participated in data collection during various time periods involving different types and frequencies of activities. The raw data were collected at a frequency of 50 Hz. This dataset comprises six different activity states.
- WISDM: The WISDM dataset was collected by the Wireless Sensor Data Mining Laboratory at Fordham University. It involves 36 volunteers who performed 6 different activities: walking, jogging, walking upstairs, walking downstairs, sitting, and standing during specific time periods. The data for each activity was recorded using the internal accelerometer sensors of smartphones placed in the pockets of the participants. The data were sampled at a rate of 20 Hz, and the dataset contains a total of 1,098,207 sample points. Each sample includes data from three accelerometer axes.
- OPPORTUNITY: The OPPORTUNITY dataset comprises a rich set of sensor information, including wearable sensors, object sensors, and environmental sensors. The wearable sensors consist of 7 inertial measurement units (IMUs), 12 3D accelerometer sensors, and 4 3D localization sensors. There are 12 object sensors and 13 switches along with 8 3D accelerometer sensors for environmental monitoring. The data collection involved four volunteers who took turns performing various daily activities within a simulated single-room apartment. The sensors recorded data related to 13 simple object-related actions, 23 object interactions, 17 gesture actions, and 5 complex activities. The data were sampled at a rate of 30 Hz. For our study, we only utilized the data related to the 17 gesture actions and exclusively used the wearable sensors.

#### 2.2.2. Data Preprocessing

Note that data are subject to issues such as missing values or anomalies due to environmental noise or data collection methods in the data collection process. Thus, data cleaning is required before data processing in the deep neural networks. Considering the special features of the investigated datasets, the detail of the cleaning methods for the previous three datasets are as follows.

- UCI-HAR: To preprocess the UCI-HAR dataset, the following steps were taken. Firstly, the sensor signals underwent noise filtering. Then, a Butterworth low-pass filter was employed to separate the accelerometer signals into body acceleration and gravity, resulting in data from six axes. In addition, data from the three-axis gyroscope were included, totaling nine channels of data. The data were processed using sliding windows with a sampling time of 2.56 s and 50% overlap, creating windows 128 samples in length.
- WISDM: The preprocessing for the WISDM dataset involved the following steps. Initially, data cleaning was conducted, followed by standardization. The dataset was then segmented using sliding windows with a length of 100 samples and 50% overlap.
- OPPORTUNITY: The data preprocessing for the OPPORTUNITY dataset involved several steps. First, the original data were cleaned, and missing data points were filled using linear interpolation. Then, sliding windows with a length of 60 samples and 50% overlap were applied to segment the dataset using selected wearable sensor data channels, resulting in 113 data channels.

## 3. Experimental Set-Up and Analysis

The proposed RMFSN model was constructed using PyTorch version 2.0.1. The experimental set-up involved a Windows 11 system with an Intel i9-13900HX CPU (Intel, Santa Clara, CA, USA) and an NVIDIA GeForce RTX 4060 GPU (NVIDIA, Santa Clara, CA, USA) with 8 GB of video memory. During the experiments, model training utilized the cross-entropy loss function to measure the disparity between the model’s predicted activity states and the true states. The adaptive moment estimation (Adam) optimization algorithm was employed to adaptively adjust the learning rate. After conducting multiple experiments, we identified relatively optimal sets of hyperparameters. Some of the hyperparameter configurations for the three datasets are provided in Table 1. Since the data channel and sampling frequencies of HAR sensors in the three investigated datasets are different, the data dimensions of the three datasets are different, and accordingly, the input sizes for the convolutional networks are distinct, as shown in Table 1, where (9, 128), (3, 100), and (113, 60) represent the (“channel”, “size of the sliding window”) of the corresponding datasets. The number of training iterations for each dataset was set to 100, and both the residual blocks and GRU layers were maintained at a depth of two layers. Additionally, the dilation rate and padding were set consistently across the datasets. The convolutional kernels in the dilated convolution layers had the same size as those in the standard convolution layers. It is important to note that the number of convolutional kernels maintained a consistent relationship with the number of feature channels.

Notice that since the RMFSN is data-driven, sufficient sensing data from human activity are required. It is time-consuming to collect and label such large amounts of raw data. Therefore, in this paper, we use the three typical public datasets UCI-HAR, WISDM, and OPPORTUNITY as the datasets for training and evaluating the proposed RMFSN. Since some data from the previous three public datasets are subject to the issues of missing values and anomalies, we performed data cleaning with the method described in Section 2.2.2 before training and evaluating the RMFSN.

In addition, since the sensing data for HAR is from different volunteers with various sensors, the number of human activity samples varies with the type of human activity, and accordingly, it is one-sided to use a single performance metric to evaluate the performance of an HAR model. Therefore, we used multiple performance metrics to evaluate the wearable sensor-based HAR models, including the proposed RMFSN. The detailed discussions and definitions of the corresponding performance metrics are described in Section 3.1.

### 3.1. Performance Metrics

The performance metrics used to investigate a deep learning-based classification method included the accuracy, precision, recall, and F1 score. These metrics could be calculated based on the confusion matrix, which served as a visual tool for illustrating the relationship between a model’s classification outcomes and the true labels. The confusion matrix consisted of four key components: true positive (TP), true negative (TN), false positive (FP), and false negative (FN).

Specifically, TP represent the number of instances where the model correctly predicted positive class samples as positive, TN represent the number of instances where the model correctly predicted negative class samples as negative, FP represent the number of instances where the model erroneously predicted negative class samples as positive, and FN represent the number of instances where the model erroneously predicted positive class samples as negative. Table 2 provides an example of a confusion matrix for a binary classification problem. The calculation methods for the above-mentioned metrics are shown in Equations (12)–(15):

(12) $$Precision=\frac{TP}{TP+FP}$$

(13) $$Recall=\frac{TP}{TP+FN}$$

(14) $$F1=\frac{2(Precison\times Recall)}{Precison+Recall}$$

(15) $$Accuracy=\frac{TP+TN}{TP+TN+FP+FN}$$

Obviously, the precision and recall are statistics specific to positive class predictions and are not well suited for HAR evaluation. While accuracy provides a comprehensive and intuitive measure by considering all samples, it is important to note that some datasets may exhibit imbalanced distributions of activity classes, which can lead to inflated accuracy, especially for the majority class. On the other hand, the F1 score is a weighted average of precision and recall, offering a more balanced assessment of model performance. Therefore, we decided to employ both the accuracy and F1 score to evaluate the model, ensuring a more robust performance assessment in light of potential class imbalance in some datasets.

### 3.2. Evaluation Method

Cross-validation (such as k-fold cross-validation and stratified cross-validation) has been widely used for evaluating the generalization performance of deep learning models [44]. However, the cross-validation method may be unsuitable for segmented time series data utilizing overlapping windows. When using sliding windows to partition data, the issue of data leakage in testing arises due to the overlap between adjacent windows allocated to different datasets [45]. The occurrence of testing data leakage can result in an overestimation of model evaluation performance, leading to potentially misleading outcomes. Thus, the model’s generalization performance in real-world scenarios may be overly optimistically estimated. Therefore, for segmented time series data, such as wearable sensor-based HAR data, a more cautious cross-validation strategy is required to mitigate this potential issue.

This paper uses leave one subject out (LOSO) cross-validation to alleviate the impact of the neighborhood bias on the performance evaluation. In particular, the validation process was determined by the number of subjects in the dataset. For each iteration, one subject’s data were selected as the test set, while the model was trained on the remaining subjects’ data. The final evaluation results were obtained by averaging the performance across all iterations.

### 3.3. Performance Analysis

Table 3 presents the performance evaluation results of our RMFSN on the UCI-HAR, WISDM, and OPPORTUNITY datasets. The average F1 scores were 98.21 (0.81)%, 97.74 (1.10)%, and 92.20 (0.25)%, and the overall accuracy was 98.13 (0.73)%, 98.35 (0.78)%, and 93.89 (0.21)%, respectively, where (·)% is the standard deviation. On the UCI-HAR dataset, activities such as “walking”, “upstairs”, “downstairs”, and “laying” exhibited consistently high performance across various metrics. Furthermore, the performance of “sitting” and “standing” activities showed some fluctuations, with a slightly higher standard deviation compared with other activities.

**Figure 8:**
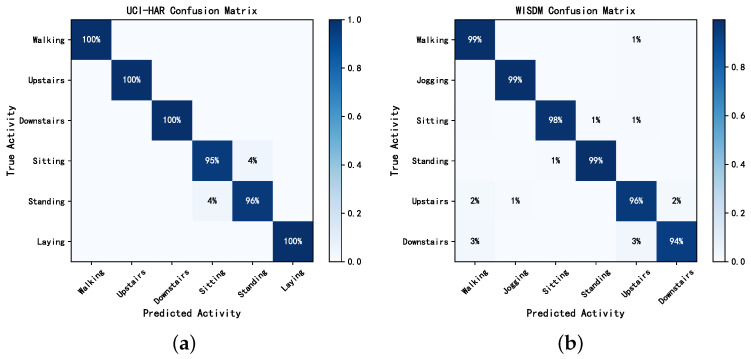
Confusion matrices for UCI-HAR and WISDM. (**a**) UCI-HAR. (**b**) WISDM.

**Figure 9:**
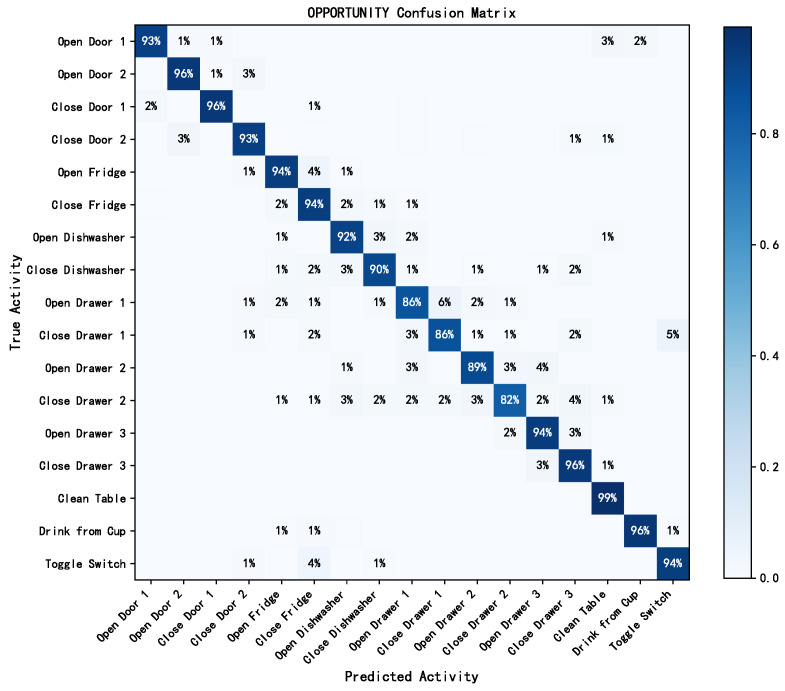
Confusion matrix for OPPORTUNITY.

Similarly, on the WISDM dataset, the first four activities, namely “walking”, “jogging”, “sitting”, and “standing”, demonstrated commendable performance across different metrics. However, the “upstairs” and “downstairs” activities exhibited slightly larger performance fluctuations. On the OPPORTUNITY dataset, most activities had average F1 scores exceeding 90%, demonstrating that the proposed model exhibited excellent discriminative capability across multiple activity classifications. A more intuitive visualization of the classification results for different activity categories is provided by the confusion matrix in Figure 8 and Figure 9.

As shown in Figure 8 and Figure 9, the RMFSN exhibited instances of mutual misclassifications on each dataset. Notably, on the UCI-HAR dataset, there was a noticeable occurrence of mutual misclassifications for the activities “sitting” and “standing”. Similarly, on the WISDM dataset, mutual misclassifications were more pronounced for the activities “upstairs” and “downstairs”. This phenomenon may be attributed to the strikingly similar data distributions of these activities within their respective datasets. Additionally, in the OPPORTUNITY dataset, suboptimal performance was observed primarily in activities related to opening and closing drawers. This could be explained by our reliance solely on wearable sensor data for this dataset without incorporating information from other types of sensors. The above phenomena also elucidates the slightly higher standard deviations observed for these activities in Table 3.

To sum up, our model demonstrated good performance on these three datasets, showcasing robust generalization capabilities.

### 3.4. Model Comparison and Visualization

To further evaluate the performance of the RMFSN model, we conducted comparisons with advanced models from recent years, using the accuracy and F1 scores as performance metrics. These studies used the same datasets to validate their model algorithms, although their data preprocessing methods varied. As such, the comparative results were not absolute but provided relative insights into the model’s performance.

Table 4 presents the comparison results with other models. Notably, our model had significantly fewer parameters than the compared methods. On the UCI-HAR dataset, our model outperformed the Bi-HAR model using a combination of the 1D-CNN and LSTM, achieving a 0.24% accuracy improvement. Compared with the ResNet model using heterogeneous convolutions, our model showed a 1.12% accuracy improvement. When compared with the GRU-INC and DMEFAM models, which also use GRUs to extract temporal features, our model demonstrated respective improvements of 1.86% and 2.13%. On the WISDM dataset, our model surpassed GTSNet, which uses group time-shift convolutions, and CapsNet, which incorporates capsule structures, with accuracy improvements of 9.48% and 1.55%, respectively. While GRU-INC achieved a slightly higher accuracy than our model (by 0.78%), it is important to note that our model had fewer parameters. On the OPPORTUNITY dataset, our model achieved an accuracy improvement of 2.34% compared with ResNet+HC, 3.52% compared with GRU-INC, and 6.42% compared with GTSNet. Likewise, the comparative results for the F1 scores did not show significant differences from the accuracy results.

**Figure 10:**
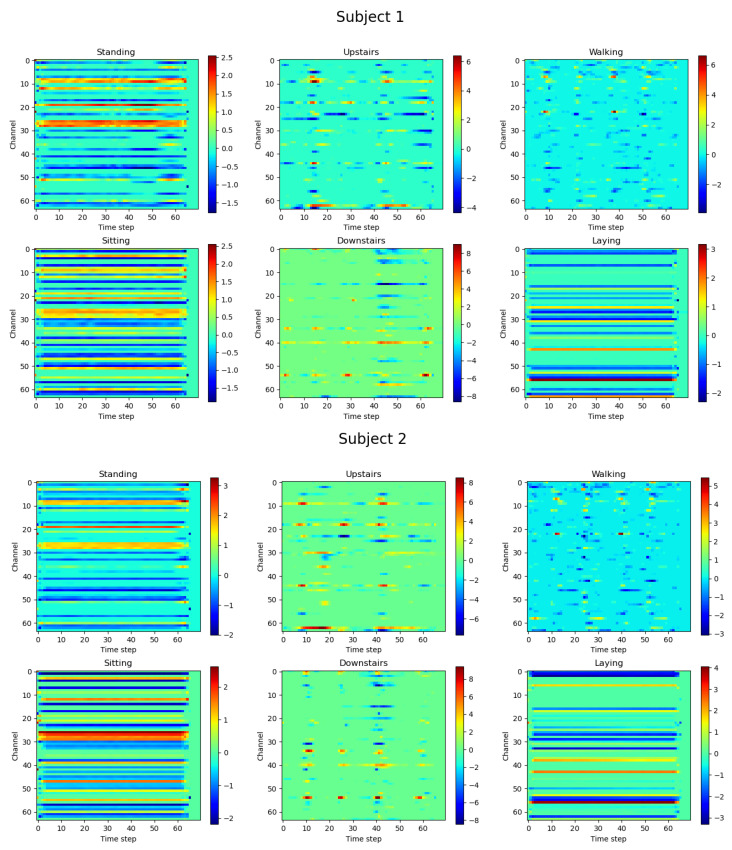
The heatmap of feature selection on the UCI-HAR dataset.

Figure 10 shows the heatmaps of feature selection on UCI-HAR for our CASB of the RMFSN to make our model more interpretable. The horizontal and vertical axes represent the time steps and feature channels, respectively. Each activity exhibited varying dependencies on features, and the RMFSN adapted its feature selection dynamically with changes in the time steps. The dynamic nature of the feature selection process underscores the model’s sensitivity to temporal variations in different activities. Meanwhile, by observing the heat maps of the two different subjects, it can be seen that the feature selection pattern of the same activity had a high degree of similarity, indicating robustness in the RMFSN’s ability to consistently identify relevant features across different individuals. This robustness underscores the model’s resilience to variations in sensor data. It can also be seen from the figure that the heat map distributions for the standing and sitting activities closely resemble each other, which may be attributed to the resemblance in sensor data for these two activities. Additionally, this observation indirectly elucidates the reasons for mutual misclassification in the confusion matrix presented in Figure 8.

### 3.5. Ablation Study

The purpose of this section’s ablation experiments is to validate the performance of each module. The experimental results assumed the use of the classifier network proposed in this paper, and except for the removed modules, all other parameters were kept unchanged. The experimental results are shown in Table 5, and it can be observed that each module had some impact on the model’s performance, with the combination of these modules leading to a more significant performance improvement. On the UCI-HAR, WISDM, and OPPORTUNITY datasets, the combination of the three modules resulted in accuracy improvements of 6.71%, 8.26%, and 7.86%, respectively, compared with the baseline. Of particular note is the significant improvement on the OPPORTUNITY dataset, which may be attributed to the dataset containing a larger number of activity categories, allowing each module to fully leverage its capabilities.

In Table 5, C, T, and M represent the channel attention shrinkage block, temporal feature extraction block, and multi-feature extraction block, respectively.

### 3.6. Impact of Evaluation Method

In order to evaluate the impact of different evaluation methods (which use different dataset partitioning methods) on the performance evaluation for HAR methods, we compared the accuracy of our proposal with three types of evaluation methods, including datasets split with various ratios, n-fold cross-validation, and LOSO. As shown in Table 6, the accuracy varied with various evaluation methods. However, the maximum deviations of the investigated methods for the UCI-HAR, WISDM, and OPPORTUNITY datasets were 0.02, 0.02, and 0.03, respectively. All are within the 95% confidence interval of LOSO, which demonstrates the robustness of our proposal for the evaluation methods.

### 3.7. Overhead Analysis

Finally, we analyzed the computation overhead of the proposed RMFSN. We used the concept of inference time, which is defined as the time duration it takes to predict a single sample, to evaluate the time complexity of the RMFSN for recognizing an activity. We conducted calculations for different samples over 300 rounds and averaged the results. The model size represents the sum of the trainable model parameters and buffer size. FLOPs denotes the number of floating-point operations involved in model inference or training and serves as a crucial metric for evaluating the computational complexity. As is illustrated in Table 7, for the wearable sensor-based HAR task, our model requires a reasonably small computation overhead and can be deployed at edge devices.

## 4. Conclusions

This paper proposed a residual multifeature fusion shrinkage network for wearable sensor-based human activity recognition. In the proposal, we used a multi-branch architecture, incorporating GRUs, dilated convolutions, and a 1D-CNN to extract diverse features. In addition, an attention mechanism was designed to enhance feature representation and crucial feature channel selection. The experimentation results, including the recognition results from trials on public datasets, ablation studies, and comparisons with existing works, demonstrated the robustness and excellent generalization capabilities of the proposal.

In future works, we will develop our dataset with more human activity samples. In addition, some techniques such as those in [47] will be introduced to mitigate potential inferences from electromagnetic radiation for improving the quality of raw data. In addition, we will improve the proposed RMFSN in the following two aspects. Firstly, we plan to add an efficient channel attention network (ECANet) to bolster the robustness of feature selection, becoming adaptive to datasets with similar or missing data. In addition, we will develop a semi-supervised learning approach to leverage a substantial amount of unlabeled data in conjunction with labeled data for model training, aiming at improving the model’s generalization ability to adapt to various scenarios.

## Figures and Tables

**Table 1:** Hyperparameter configurations.

Hyperparameter	UCI-HAR	WISDM	OPPORTUNITY
Input size	(9, 128)	(3, 100)	(113, 60)
Epochs	100	100	100
Batch size	155	256	256
Learning rate	$5.0\times 10^{-4}$	$1.0\times 10^{-3}$	$1.0\times 10^{-3}$
Filters and feature	64	75	110
Residual block	2	2	2
GRU layer	2	2	2
Stem kernel	4	5	7
Dilated kernel	(4, 2)	(3, 2)	(3, 5)
Dilated rate	(1, 2, 4, 6)	(1, 2, 4, 6)	(1, 2, 4, 6)
padding	(2, 4, 8, 10)	(2, 4, 8, 10)	(2, 4, 8, 10)

**Table 2:** Confusion matrix for binary classification.

	Predicted	Positive (1)	Negative (0)
True			
Positive (1)		TP	FN
Negative (0)		FP	TN

**Table 3:** The performance of the proposed model on UCI-HAR, WISDM, and OPPORTUNITY datasets (standard deviations are shown in parentheses).

Performance		Precision (%)	Recall (%)	F1-Score (%)
UCI-HAR	Walking	99.52 (0.26)	99.52 (0.26)	99.52 (0.26)
	Upstairs	99.59 (0.32)	99.62 (0.12)	99.60 (0.12)
	Downstairs	99.71 (0.18)	100 (0)	99.85 (0.10)
	Sitting	95.83 (1.53)	94.84 (2.52)	95.10 (2.18)
	Standing	96.08 (1.23)	96.00 (1.81)	96.04 (1.61)
	Laying	99.51 (0.33)	100 (0)	99.75 (0.20)
WISDM	Walking	98.54 (0.38)	99.16 (0.54)	98.95 (0.87)
	Jogging	99.67 (0.20)	99.48 (0.25)	99.57 (0.18)
	Sitting	99.32 (0.38)	98.20 (1.38)	98.76 (0.55)
	Standing	98.98 (0.41)	99.12 (0.53)	99.05 (0.70)
	Upstairs	94.42 (2.21)	95.86 (2.35)	95.13 (1.54)
	Downstairs	96.37 (1.71)	94.03 (3.47)	95.19 (2.16)
OPPORTUNITY	Open Door 1	97.51 (1.10)	92.82 (1.30)	95.10 (1.20)
	Open Door 2	95.85 (1.91)	96.03 (0.56)	95.93 (1.04)
	Close Door 1	97.77 (0.85)	95.89 (0.56)	96.82 (0.67)
	Close Door 2	91.93 (4.30)	93.12 (0.67)	92.48 (2.43)
	Open Fridge 1	93.56 (2.02)	93.86 (0.59)	93.70 (1.27)
	Close Fridge 2	87.04 (4.28)	94.08 (0.51)	90.38 (2.45)
	Open Dishwasher 1	90.13 (3.24)	91.88 (1.23)	90.94 (1.05)
	Close Dishwasher 2	92.04 (3.43)	89.48 (1.40)	90.69 (1.48)
	Open Drawer 1	84.42 (5.01)	85.49 (1.67)	84.48 (2.24)
	Close Drawer 1	88.27 (4.33)	86.30 (2.71)	87.16(1.94)
	Open Drawer 2	92.00 (2.50)	88.77 (1.68)	90.27 (2.97)
	Close Drawer 2	90.72 (2.72)	81.67 (0.80)	85.94 (1.53)
	Open Drawer 3	91.52 (4.05)	94.35 (0.78)	92.87 (2.15)
	Close Drawer 3	89.52 (2.81)	95.66 (0.44)	92.47 (1.61)
	Clean Table	94.51 (1.80)	99.28 (0.43)	96.83 (0.97)
	Drink from Cup	99.19 (0.30)	96.46 (0.44)	97.80 (0.19)
	Toggle Switch	93.10 (2.14)	94.00 (0.41)	93.54 (1.27)

**Table 4:** Performance comparison.

Dataset	Approach	Accuracy (%)	F1-Score (%)	No. of Parameters
UCI-HAR	Bi-HAR [29]	97.89	96.47	15,017,152
	ResNet+HC [30]	97.01	-	0.42 M ^1^
	GRU-INC [32]	96.27	96.26	666,112
	DMEFAM [34]	96.00	95.80	1.6 M ^1^
	RMFSN	98.13	98.21	239,846
WISDM	GRU-INC [32]	99.13	99.12	661,486
	DMEFAM [34]	97.90	97.00	1.55 M ^1^
	CapsGaNet [35]	96.80	96.20	-
	GTSNet [46]	88.87	88.60	-
	RMFSN	98.35	97.74	303,939
OPPORTUNITY	ResNet+HC [30]	91.55	-	1.55 M ^1^
	GRU-INC [32]	90.37	90.05	723,728
	GTSNet [46]	87.47	87.40	-
	RMFSN	93.89	92.20	524,482

^1^ The M in the table represents millions of units.

**Table 5:** Ablation results.

Model	Accuracy (%)		
	UCI-HAR	WISDM	OPPORTUNITY
Without C	94.42	95.14	91.05
Without T	95.28	96.12	91.64
Without M	95.64	95.87	91.12
Without T and M	93.12	92.02	90.16
Without C, T, and M	91.42	90.07	86.03
RMFSN	98.13	98.35	93.89

**Table 6:** Accuracy under various evaluaton methods.

Ratio	Accuracy		
	UCI-HAR	WISDM	OPPORTUNITY
6:1:3	98.38	96.57	91.33
7:1:2	97.65	98.42	94.47
8:1:1	97.24	97.62	93.42
5 fold	97.85	98.14	94.68
10 fold	96.12	97.79	92.86
LOSO	98.13	98.35	93.89

**Table 7:** The overhead of the proposed model.

Dataset	Inference Time (ms)	Model Size (M)	FLOPs (M)	No. of Parameters
UCI-HAR	2.52	0.92	15.40	239,846
WISDM	3.12	1.15	15.34	303,939
OPPORTUNITY	3.54	1.98	18.57	524,482

## Data Availability

Data are contained within the article.

## Abbreviations

The following abbreviations are used in this manuscript:

HAR	Human activity recognition
RMFSN	Residual multifeature fusion shrinkage network
TFEB	Temporal feature extraction block
GRU	Gated recurrent unit
MSFEB	Multi-scale feature extraction block
CASB	Channel attention shrinkage block
GAP	Global average pooling
GMP	Global max pooling
LOSO	Leave one subject out
